# Identifying Influential Factors in the Discussion Dynamics of Emerging Health Issues on Social Media: Computational Study

**DOI:** 10.2196/17175

**Published:** 2020-07-28

**Authors:** Lida Safarnejad, Qian Xu, Yaorong Ge, Arunkumar Bagavathi, Siddharth Krishnan, Shi Chen

**Affiliations:** 1 College of Computing and Informatics University of North Carolina at Charlotte Charlotte, NC United States; 2 School of Communications Elon University Elon, NC United States; 3 Oklahoma State University Oklahoma, OK United States; 4 College of Health and Human Services University of North Carolina at Charlotte Charlotte, NC United States

**Keywords:** social media, infodemiology, infoveillance, infodemic, health emergency, tweeting dynamics, events detection, online influentials, Zika, public engagement

## Abstract

**Background:**

Social media has become a major resource for observing and understanding public opinions using infodemiology and infoveillance methods, especially during emergencies such as disease outbreaks. For public health agencies, understanding the driving forces of web-based discussions will help deliver more effective and efficient information to general users on social media and the web.

**Objective:**

The study aimed to identify the major contributors that drove overall Zika-related tweeting dynamics during the 2016 epidemic. In total, 3 hypothetical drivers were proposed: (1) the underlying Zika epidemic quantified as a time series of case counts; (2) sporadic but critical real-world events such as the 2016 Rio Olympics and World Health Organization’s Public Health Emergency of International Concern (PHEIC) announcement, and (3) a few influential users’ tweeting activities.

**Methods:**

All tweets and retweets (RTs) containing the keyword Zika posted in 2016 were collected via the Gnip application programming interface (API). We developed an analytical pipeline, EventPeriscope, to identify co-occurring trending events with Zika and quantify the strength of these events. We also retrieved Zika case data and identified the top influencers of the Zika discussion on Twitter. The influence of 3 potential drivers was examined via a multivariate time series analysis, signal processing, a content analysis, and text mining techniques.

**Results:**

Zika-related tweeting dynamics were not significantly correlated with the underlying Zika epidemic in the United States in any of the four quarters in 2016 nor in the entire year. Instead, peaks of Zika-related tweeting activity were strongly associated with a few critical real-world events, both planned, such as the Rio Olympics, and unplanned, such as the PHEIC announcement. The Rio Olympics was mentioned in >15% of all Zika-related tweets and PHEIC occurred in 27% of Zika-related tweets around their respective peaks. In addition, the overall tweeting dynamics of the top 100 most actively tweeting users on the Zika topic, the top 100 users receiving most RTs, and the top 100 users mentioned were the most highly correlated to and preceded the overall tweeting dynamics, making these groups of users the potential drivers of tweeting dynamics. The top 100 users who retweeted the most were not critical in driving the overall tweeting dynamics. There were very few overlaps among these different groups of potentially influential users.

**Conclusions:**

Using our proposed analytical workflow, EventPeriscope, we identified that Zika discussion dynamics on Twitter were decoupled from the actual disease epidemic in the United States but were closely related to and highly influenced by certain sporadic real-world events as well as by a few influential users. This study provided a methodology framework and insights to better understand the driving forces of web-based public discourse during health emergencies. Therefore, health agencies could deliver more effective and efficient web-based communications in emerging crises.

## Introduction

### Background

Social media platforms, such as Twitter and Facebook, are attracting a growing number of people with diverse demographic characteristics to share and obtain information on the web. As a result, these platforms have become one of the main targets for practitioners and decision makers across various fields to understand public opinion and, at the same time, disseminate information to the public [1-17]. Many public health agencies and organizations, such as the US Centers for Disease Control and Prevention (CDC), are active on Twitter and other social media platforms as the main channels of communication with the general public, especially during health emergencies such as the 2014 Ebola and 2016 Zika outbreaks. The CDC has 67 officially associated Twitter accounts that cover a wide variety of health- and disease-related topics. In 2016, when Zika caused 5168 confirmed noncongenital cases in 50 states and the District of Columbia in the United States, and a much higher case number across US territories [6], former CDC director Dr Tom Frieden was active on Twitter and hosted live Twitter chats with the general public [18], including a 1-hour live chat session with the public regarding Zika in February 2016.

Nevertheless, there are multiple challenges in utilizing social media platforms as an effective channel of communication. A considerable percentage of users are unfamiliar with the emerging health issue. At the same time, user-posted content does not go through any rigorous fact-checking process, making room for misinformation to take advantage of such a situation. During the 2016 Zika epidemic, despite the CDC’s prominent web presence and efforts, inaccurate information regarding Zika proliferated on social media and outperformed CDC (and other legitimate sources such as the World Health Organization [WHO]) by a large margin [7]. Uncertainty about the root cause and transmission route of this virus gave room for the proliferation of rumors and misinformation [19,20].

In addition to the problem of misinformation propagation, the rhetorical aspect of a message, or in other words, crafting it based on the needs and perception of audiences is a critical challenge [21,22]. Studies have shown a substantial topic discrepancy between public concern and the CDC’s response to Zika on Twitter [8,9,20,23]. More specifically, the general public was more concerned about the transmission routes of Zika and effective prevention methods, whereas the CDC focused on symptoms to educate the public [24,25]. Glowacki et al [25] argued that this could be seen as failure of the CDC to identify what kind of information the public was looking for and respond accordingly or it could be an on-purpose attempt by the CDC to redirect public attention to what the CDC believed to be more important during the epidemic.

In addition, one important yet overlooked issue in utilizing social media platforms as a communication mechanism with the public is the low rate of user engagement (measured by the number of retweets [RTs] and replies), where social media is an interactive platform for public engagement and interaction [26], in addition to one-directional news outlets [10,27,28]. To better engage the public, it is essential to recognize critical factors that are directing and driving the general discussion dynamics on social media. Such factors can be discovered by observing and analyzing the public’s tweeting behaviors on social media [29,30]. Learning about these factors can help health agencies to accurately predict shifts in the public’s concern about the health issue and provide the public with useful information accordingly. As a result, systematically collecting and analyzing data related to public discourse of emerging health issues on social media, also referred to as digital public health surveillance, infodemiology, or infoveillance [31], is essential for understanding public concerns and disseminating useful information correspondingly.

### Objectives

In this study, we aimed to identify important factors that could potentially drive tweeting dynamics in the 2016 Zika epidemic. We collected and comprehensively analyzed all Zika-related English tweets posted during 2016. We further proposed and evaluated the following 3 testable hypotheses (H):

1. H1: The observed overall tweeting dynamics of Zika was associated with and influenced by the underlying Zika epidemic, defined as the number of case counts per day, especially in the United States.
2. H2: The tweeting dynamics of Zika was associated with and influenced by a few real-world critical events, other than the continuous Zika epidemic.
3. H3: The tweeting dynamics of Zika were driven by a few highly influential users (colloquially referred to as influentials hereafter), which led to the public discourse of Zika on Twitter.

## Methods

### Data Acquisition

We requested and retrieved more than 6 million English tweets, including the keyword *Zika* from January 1 to December 31, 2016, via the Gnip application programming interface (API) through the Data Science Initiative (DSI), University of North Carolina Charlotte. All associated metadata with these tweets, such as RT counts, posted time, and the verification status of tweeting/retweeting ID, were also included in the data set. This data set represented the complete public discourse about Zika in English and was therefore not as prone to potential selection bias as the common 10% sample provided by the common Twitter API. Therefore, the data set in this study was able to provide an unbiased and comprehensive depiction of the public’s discourse of Zika, the most discussed health topic in 2016 on a major social media platform.

In addition to web-based Twitter data, the complete time series of confirmed noncongenital Zika case counts in 2016 in the United States were obtained from the CDC’s database [32]. Both domestic cases (cases in 50 states and District of Columbia) and all cases combined (cases in 50 states, District of Columbia, and overseas territories such as Puerto Rico, Virgin Island, Guam) were acquired.

### Association Between the United States Zika Epidemic and Tweeting Dynamics

Zika case counts in 2016 were retrieved from the CDC [32] and then downscaled into standardized daily counts using the cubic spline interpolation method [27]. The time series of the downscaled daily case counts was then compared with daily Zika-related tweet counts. As both time series (cases and tweets) had the same daily resolution and the same length of 366 days, a cross-correlation function (CCF) was applied to quantify potential association and time lags between the two time series. CCF measured the temporal similarity between the two time series, as shown in equation (1). The significance level was set at 0.2 by default in the analytical package in this study. In general, larger absolute values of cross-correlation at time lag *L* indicate a stronger association between the two time series. Both domestic US Zika cases and all US Zika cases were compared with Zika-related tweet counts in each of the four quarters of 2016 as well as during the entire 2016 period:


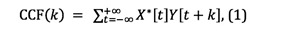


In addition, mutual information (MI) between Zika case counts and Zika-related tweets in each of the four quarters as well as in 2016 was quantified to further evaluate the mutual dependence of the two time series. MI was calculated as the expected value of the pointwise MI (PMI) of the two time series. PMI measured the level of dependency between 2 observations [33]. PMI between *X* and *Y* was calculated using equation (2):


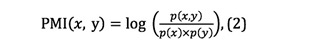


where *p* (.) is the probability function. The 2 observations that had a high PMI value were strongly associated with each other. In other words, they frequently co-occurred. The average dependency or MI between the 2 random variables *X* and *Y* was then calculated using equation (3):


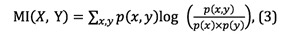


CCF provided an overview of the association between real-world Zika case counts and tweeting activities regarding Zika over a period. A CCF above 0.05 indicated a strong association between the two time series. MI further quantified this association with a value. These two approaches complemented each other.

### Association Between Critical Events and Tweeting Dynamics

**Figure 1:**
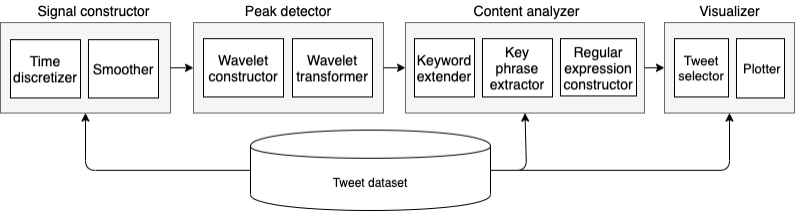
EventPeriscope analytical pipeline.

Health emergencies, such as the Zika epidemic, would never occur in isolation and almost always be intermingled with other health, social, societal, and political events in the real world. We suggest that related and sometimes unrelated real-world events could be potential driving forces of Zika discussions on social media. Unlike the time series of daily Zika case counts, these real-world events were much more discrete and sporadic. Here, we evaluated the second hypothesis (H2) such that Zika-related tweeting activities were substantially influenced by sporadic real-world events. We adopted the definition of an event provided by Hasan et al [18] stating, “An event, in the context of social media, is an occurrence of interest in the real world which instigates a discussion on the event-associated topic by various social media users, either soon after the occurrence or, sometimes, in anticipation of it.” We developed an analytical pipeline, EventPeriscope, to explore and quantify the impact of real-world events on the tweeting dynamics of a specific topic (eg, Zika in this study) and to evaluate H2. Figure 1 demonstrates the 4 main components of the EventPeriscope pipeline: signal constructor, peak detector, content analyzer, and visualizer.

The signal constructor module modeled the number of daily tweets about Zika as a signal to characterize its temporal changes. If a particular real-world event had a significant influence on Twitter discussions, we would expect to see a peak in the time of the event or close to it. Therefore, a sudden rise in the signal indicated the high engagement of Twitter users, which might be linked to a potential real-world event. To identify these peaks, the peak detector module applied the wavelet transform to detect peaks in the constructed signal from the signal constructor module. Nevertheless, a peak at or around the time of a real-world event was only a necessary but not sufficient condition to conclude that the event was the main driver of the rise in the number of tweets. Overlap of the event and peak of Zika-related tweeting might be coincidental. It would be critical to demonstrate that Zika-related tweets around the event were actually regarding that event. To confirm this relevance, the content analyzer module then analyzed textual contents of the tweets around the real-world event to extract all key phrases that were relevant to the event. Then, the content analyzer created regular expression (regex) rules to automatically capture all variations and combinations of these key phrases. Finally, the visualizer module compared all tweets in the data set against the constructed event-specific regex rules and constructed a new time series from the matched tweets as the signal of a specific event related to Zika. It helped to understand how discussions spanned around the event in a wider time window. To illustrate how all 4 modules worked synergistically in EventPeriscope, we provided case studies of critical real-world events and their impact on Zika-related tweeting dynamics.

**Case Studies of Critical Events**

Real-world events could be categorized into 2 dichotomized and mutually exclusive types [34]: (1) planned (ad hoc) events that people expected in advance, such as the 2016 Rio Olympics; (2) unplanned (posthoc) events that people would not know beforehand, contrary to planned events. An example of unplanned events was the WHO’s Public Health Emergency of International Concern (PHEIC) announcement about Zika on February 1, 2016. In the next section, we have discussed methodological differences in exploring planned (Rio Olympics 2016) and unplanned (WHO-PHEIC) events in detail. Planned events might increase their presence in tweeting around the event, but it could be mentioned throughout the entire year because people were well aware of it beforehand. Unplanned events, however, would not be mentioned in tweets until their occurrence in the real world. In the next section, we examine the impact of these 2 types of real-world events on Twitter discussion dynamics.

**Unplanned Event: World Health Organization’s Public Health Emergency International Concern Announcement**

On February 1, 2016, the director-general of WHO, Margaret Chan, declared a PHEIC of a potential Zika pandemic [35]. In this statement, in addition to raising concerns over the linkage of Zika with microcephaly and other neurological disorders, the WHO provided travel advice in Zika-impacted regions. The WHO-PHEIC announcement was an unplanned event, and the general public did not have any previous knowledge of its occurrence. Therefore, it should only influence tweets posted after the PHEIC announcement. We used EventPeriscope to quantify the influence of the WHO-PHEIC event on Zika-related tweeting as follows.

First, a signal was constructed from all posted Zika-related tweets, which is hereafter referred to as the main tweet signal. The main tweet signal peak in the entire 2016 period occurred almost immediately after the WHO-PHEIC event on day 32 (February 1, 2016), indicating a potential and strong correlation between the event and Zika-related tweeting. Textual contents of tweets were then analyzed to verify the association between Zika-related tweets and the WHO-PHEIC announcement. The set of tweets posted in a 2-day interval, the day of the WHO-PHEIC announcement (February 1) and 1 day after (February 2), were used as the input of the content analyzer (CA) module to construct a regex rule describing the WHO-PHEIC event. In addition, this module was given a set of 2 additional keywords, *WHO* and *PHEIC*, relevant to the WHO-PHEIC announcement event. To find other relevant keywords, the keyword extractor in the CA module used PMI, which was discussed in the previous section, and calculated PMI values between each of these 2 keywords and all the keywords extracted from the input signal. The new keywords were then sorted in descending order based on PMI values, and those with the highest PMI values were selected. In this study, we selected the top 6 keywords from the list.

Using this approach, the additional set of keywords included *emergency*, *public*, *international*, *global*, *world*, and *health*. A single word within a tweet was usually not adequate to reveal the topic of the content. Therefore, to consider the context of a tweet and obtain a more accurate result, the key phrase extractor uses these keywords to synthesize key phrases describing the event. We defined a key phrase as a noun phrase that contained at least one of the keywords. The key phrases relevant to WHO-PHEIC were *public health emergency*, *global emergency*, *international emergency*, and *world health*. On the basis of these key phrases, a regex rule was crafted. Using a similar approach, another regex rule was generated to capture Zika-related tweets relevant to WHO, regardless of whether it was related to WHO-PHEIC. Finally, the visualizer module compares all tweets in the input data set with these regex rules and generated 2 output signals: one for WHO-PHEIC and the other for WHO.

**Planned Event: RIO2016**

The Rio 2016 Olympic Games were held from August 5 to 21, 2016, in Rio de Janeiro, Brazil, amid global concerns about the Zika outbreak. In November 2015, Brazilian authorities declared a national public health emergency due to a high Zika incidence [26]. As RIO2016 was a planned event, we expected to see tweeting about Zika and RIO2016 before its opening. The CA module of the EventPeriscope pipeline was initialized with tweets posted from August 4 to 6 (days 217 to 219) within plus or minus a 1-day window of the RIO2016 opening. Then, a regex rule was generated to detect the co-occurrence of the Zika and Rio Olympics topics in Twitter discussions. The final keywords and key phrases were *Rio*, *Olympics*, *Rio2016*, *2016 Olympics*, and *Rio Olympics*.

**Association Between Web-Based Influentials and Zika-Related Tweeting Dynamics**

In this part of the study (H3), we hypothesized that a few influentials on Twitter made a substantial contribution in driving the tweeting dynamics, that is, a noticeable sudden rise in the number of tweets. To evaluate this hypothesis, we defined 4 different types of web-based influentials in 2 major categories: active influentials who posted a large number of original tweets about Zika (top tweeter [TT]) and who retweeted a lot about Zika from other accounts’ posts (top retweeter [TR]). These users actively disseminated Zika-related information to the public. In addition, influentials on social media could be passive as well: whose original posts were retweeted a lot (top received retweets [TRRT]) and who received many mentions (@_userID) from other Twitter users (top mentioned [TM]). These passive influentials, on the other hand, were more reflective of public perception and engagement of Zika discussions on Twitter. We ranked and selected the top 100 users in each of these 4 influential groups: TT, TR, TRRT, and TM. The tweeting dynamics of each user in the TT, TRRT, and TM groups and the retweeting dynamics of each influential in the TR group were extracted as their respective time series signals. These tweeting/retweeting signals were then aggregated and compared with the overall tweeting dynamics using a CCF in each quarter of 2016 as well as the entire year. This step tested the group-level association between types of influential and overall Zika-related tweeting dynamics. In addition, we derived the time lag between each influential’s tweeting (or retweeting) dynamics and the main tweet signal to test if these tweeting activities of influentials preceded the overall tweeting dynamics. This step was critical to further reveal if these influentials actually initiated an increasing number of Zika-related tweets, or the other way around, that is, these influentials were actually following and catching up with the general trend on Twitter. We also examined the overlap between the 4 groups of influentials by calculating the intersection of any 2 sets of influentials. This would reveal if certain influential group(s) on Twitter would also be influential in other ways. In particular, we wanted to identify influentials who were both actively disseminating information to the public (ie, in TT or TR groups) and passively receiving attention from the general public on social media (ie, in TRRT or TM groups).

The work was carried out in Python 3.7 (Python Software Foundation) for data retrieving and EventPeriscope pipeline construction. In addition, *R* 3.3.1 was used for the statistical analyses. All codes associated with this study were freely available upon request.

## Results

### Descriptive Results of the Zika-Related Tweeting Dynamics

A total of more than 6 million English tweets with the keyword *Zika* posted during 2016 were retrieved, of which approximately 4 million were original posts, and the remaining were RTs. More than 70% of the original posts received no RT at all, and only 2% of tweets received at least five RTs. The Gini coefficients of the number of RTs were 0.74 and 0.98 for all original tweets and original tweets that received RTs, respectively. This indicated a very high heterogeneity in the potential influence of individual tweets on social media.

### Association Between the Zika Epidemic and Tweeting Dynamics

**Figure 2:**
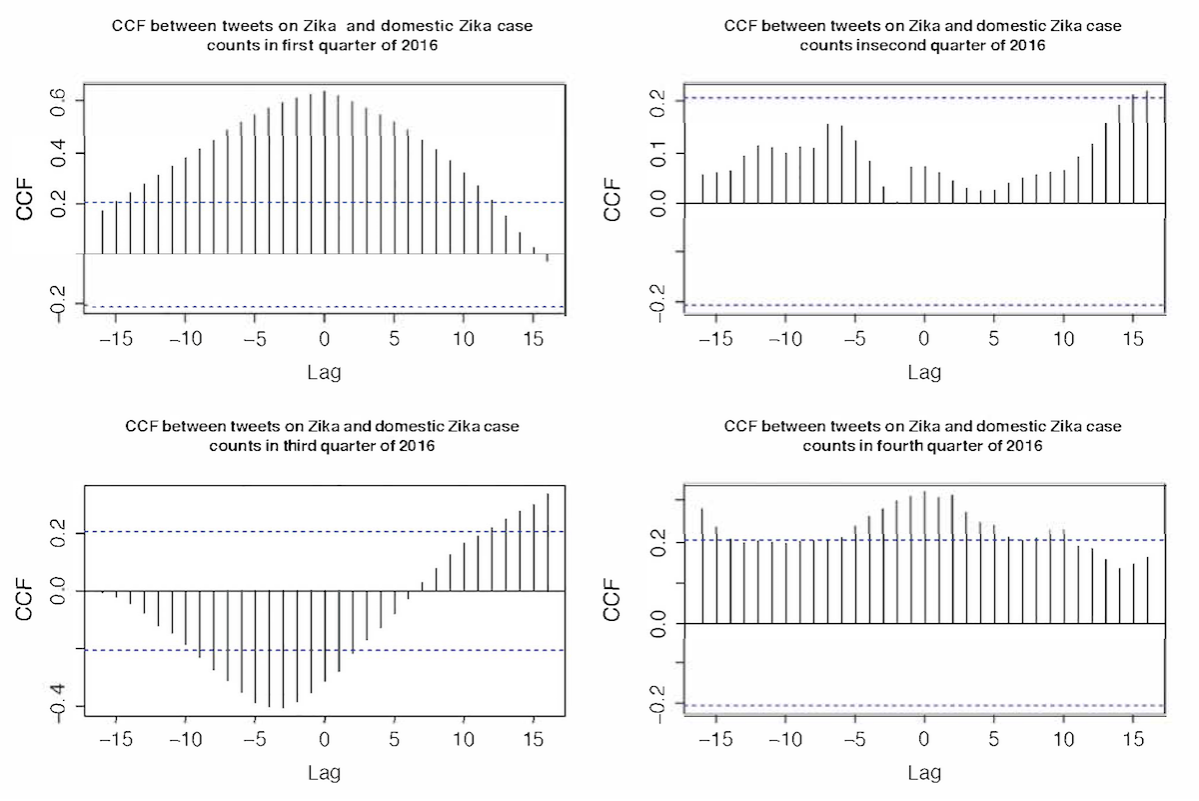
Cross-correlation function between Zika case counts in the domestic United States and tweet counts in 2016. CCF: cross-correlation function.

**Figure 3:**
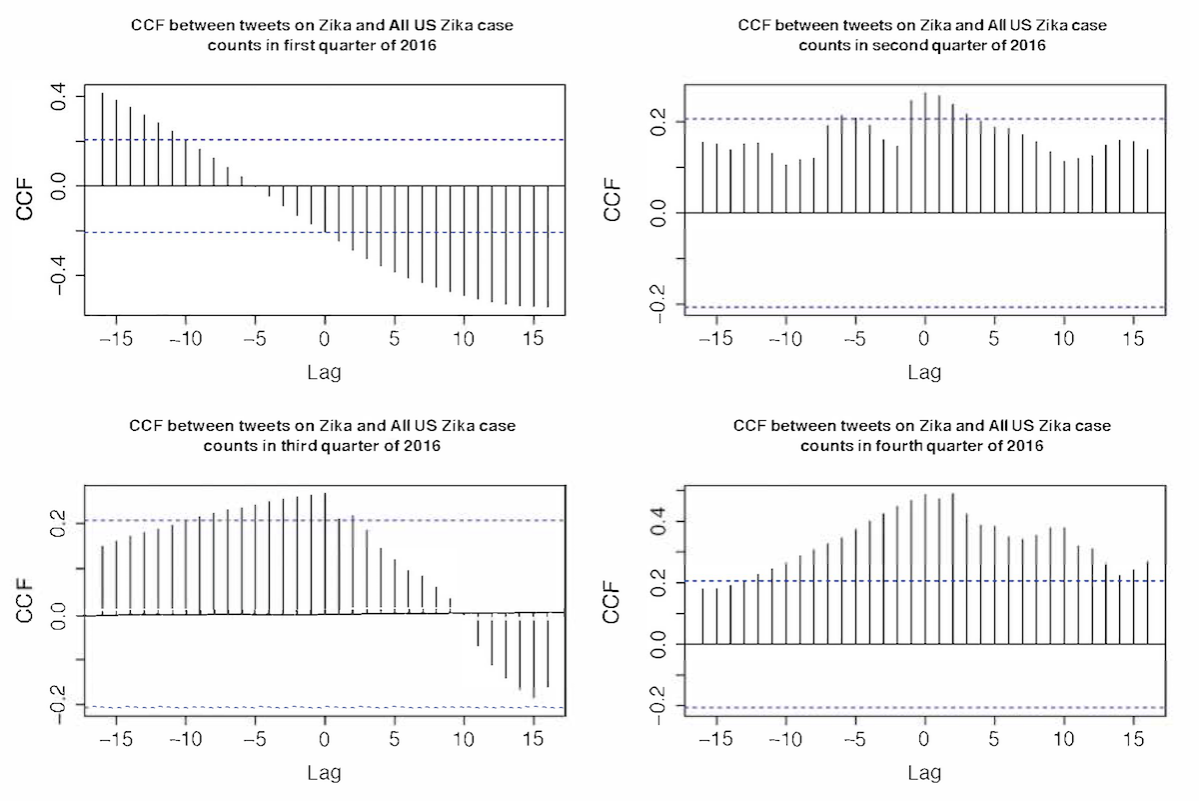
Cross-correlation function between Zika case counts in the domestic United States plus overseas territories and tweet counts in 2016. CCF: cross-correlation function.

No significant cross-correlation between domestic Zika cases in the United States and overall discussion dynamics on Twitter was observed in any of the four quarters in 2016 (Figure 2). Although in the first quarter, the CCF was substantially above the threshold, it was distributed almost normally around 0, indicating a lack of time lag between the domestic Zika case and Twitter discussion dynamics. Similarly, no substantial cross-correlation between all Zika cases in the United States (including overseas territories) and Twitter discussion dynamics was prominent in any quarter in 2016 (Figure 3). For all Zika cases, including overseas territories, the highest cross-correlation occurred in the fourth quarter, which was different from the domestic case with the highest correlation in the first quarter. These results demonstrated that Zika-related tweeting dynamics were decoupled from the actual disease epidemic in the United States, indicating that the underlying Zika epidemic did not substantially influence the Zika discussion on Twitter. In fact, the highest peak of Zika-related tweeting occurred around February 1, 2016, where the case counts were low in the United States, both domestically and overseas. Therefore, such prominent peaks in Zika-related tweeting dynamics should be explained by other driving forces than the actual Zika case counts. MI between the two time series, as shown in Table 1, was lower in 2016 but substantially higher in each quarter. The highest MI occurred in the second quarter when the number of new Zika cases was the highest in 2016. However, most Zika-related tweets were tweeted in the first quarter of 2016.

**Table 1:** Mutual information between Zika case counts and tweet counts in the United States in 2016.

Case counts in the United States	Quarter 1	Quarter 2	Quarter 3	Quarter 4	2016
Domestic	2.15	3.40	2.99	2.51	1.89
Domestic and overseas territories	2.93	3.23	2.45	2.64	1.83

### Association Between Sporadic Critical Events and Zika-Related Tweeting Dynamics

**Figure 4:**
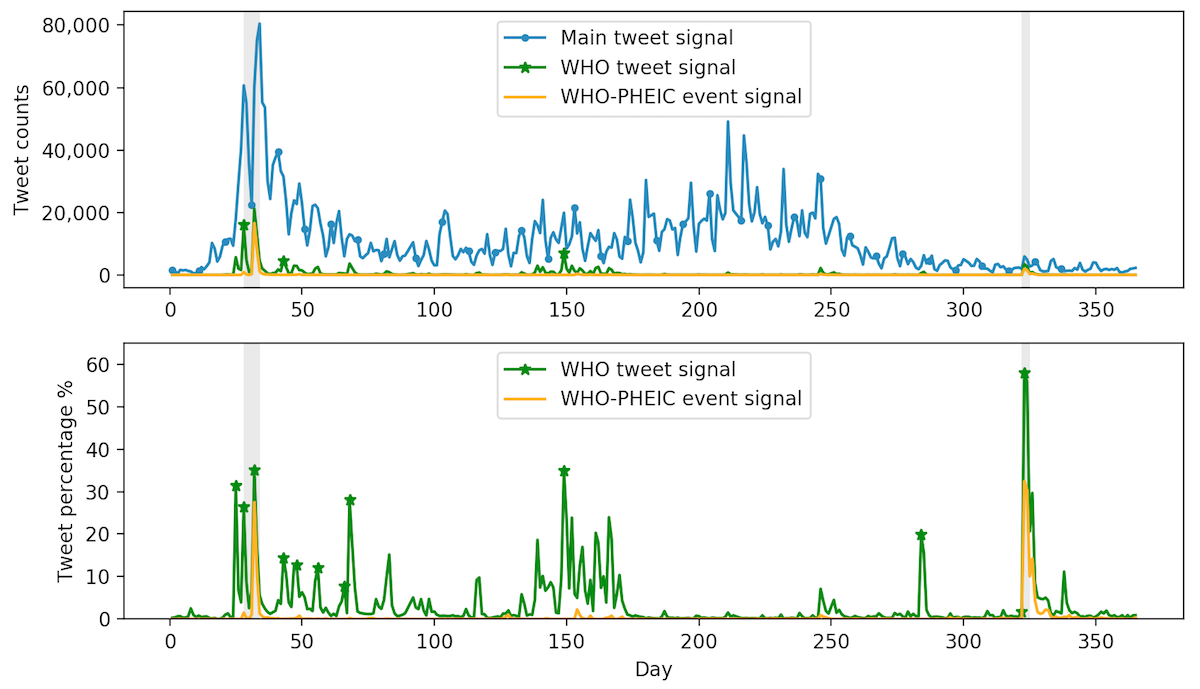
Signals of the main Zika-related tweets, WHO tweets, and WHO-PHEIC tweets. WHO: World Health Organization; PHEIC: Public Health Emergency of International Concerns.

The peaks of Zika-related tweets were not synchronized with peaks of Zika counts, as discussed in the previous section. In fact, a large number of Zika-related tweets were associated with a few sporadic real-world events. The association between Zika-related tweets and the unplanned real-world event WHO-PHEIC announcement is shown in Figure 4. WHO and WHO-PHEIC tweets were subsets of all Zika-related tweets. The upper panel of Figure 4 is the absolute number of tweet counts. The blue signal shows all Zika-related tweets in 2016. The green and orange signals represent WHO and WHO-PHEIC signals, respectively. The lower panel of Figure 4 shows the percentage of WHO and WHO-PHEIC tweets relative to all Zika-related tweets. If a tweet had both keywords/key phrases of WHO and PHEIC, then the same tweet would be included in both categories. PHEIC- and WHO-related tweets had a high overlap (>50%), indicating the substantial impact of the WHO-PHEIC announcement on public discourse on social media.

The keyword *WHO* had a strong presence in Zika-related tweeting throughout the first two quarters of 2016. There was a sudden rise in the number of tweets between days 31 and 32 of 2016 (Figure 4); the number of Zika-related tweets increased drastically from 1481 on day 31 (January 31) to 21,171 on day 32 (February 1), when the WHO announced the Zika epidemic as PHEIC. On February 1, 2016, 35% of all Zika-related tweets were relevant to WHO and 27% were about the announcement of PHEIC. This announcement also caused cascading public announcements in countries such as Brazil, Honduras, and the United States. The highest number of tweets (92,000) posted on a single day regarding Zika was observed on day 34, just 2 days after the WHO-PHEIC announcement. Therefore, the unplanned WHO-PHEIC announcement was the driving force of the largest peak of Zika-related tweeting dynamics in 2016. It is worth noting that the discussion about the PHEIC started on January 28, when the director-general of WHO announced that she convened the International Health Regulations emergency committee and would have a meeting on February 1 [35]. In addition to this peak, the WHO-PHEIC signal had another prominent peak around day 323 (November 18, 2016; Figure 4). On November 18, 2016, about 32% of the Zika-related tweets were related to WHO-PHEIC because WHO declared that the Zika epidemic was no longer a PHEIC on that specific day. Therefore, our EventPerisope analytical pipeline was effective in identifying and evaluating the impact of real-world events on tweeting dynamics.

**Figure 5:**
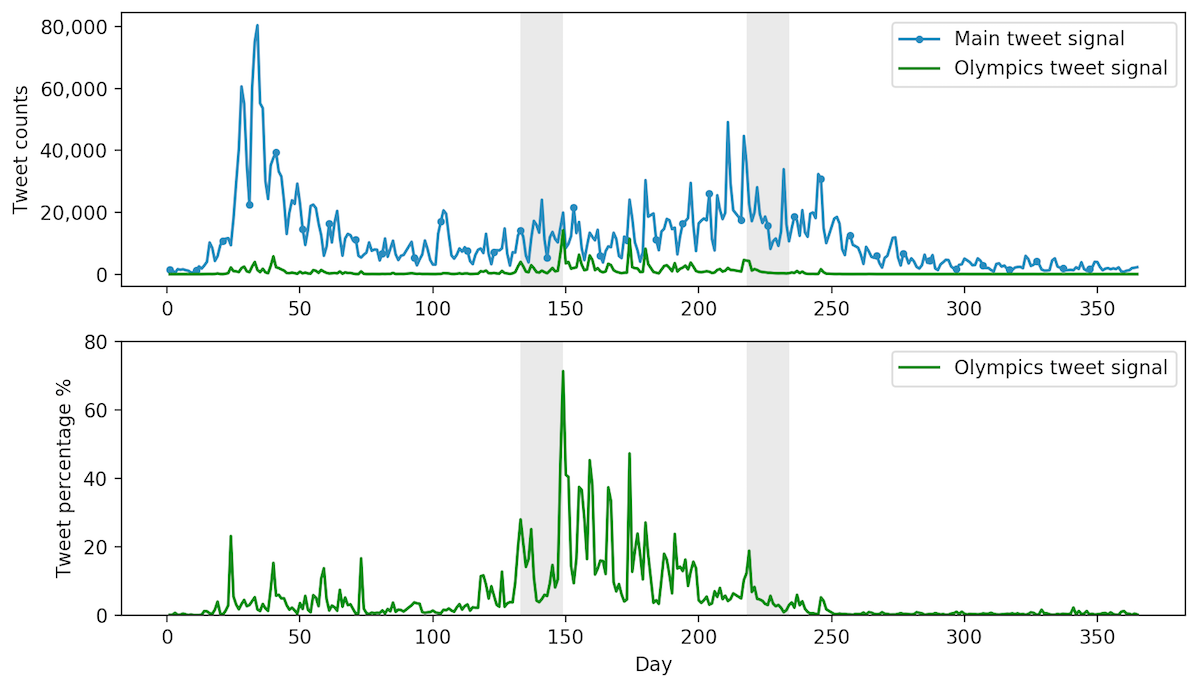
Signals of the main Zika-related tweets and RIO2016 tweets.

The association between the planned event, RIO2016, and the peaks of Zika-related tweeting are shown in Figure 5. The upper panel shows the absolute number of tweet counts. The blue and green signals showed all Zika-related tweets and RIO2016 Olympics tweets in 2016, respectively. The lower panel shows the percentage of RIO2016-related tweets relative to all Zika-related tweets. In general, discussions about Zika and the RIO2016 Olympics started from the beginning of 2016 all the way through a few days after the Olympics ended. In other words, although the event of the RIO2016 Olympics only lasted for 2 weeks, the discussion of this event with regard to Zika went on throughout the entire year because the Olympics was a planned event. Specifically, on its opening ceremony day (August 5) and on the next day, 12% and up to 18% of all Zika-related tweets were related to RIO2016, respectively.

In addition, RIO2016 had a prominent presence in other noticeable peaks of the Zika-related tweeting signal. For example, RIO2016 constituted 71% of all Zika-related tweets on day 149 (May 28). Our further investigation revealed that on day 133 (May 12), a researcher started the debate that RIO2016 should be canceled or at least postponed amid concerns of the Zika outbreak [36]. However, on day 149 (May 28), the WHO released a statement [35] explaining that it was not necessary to take such an action. Owing to the WHO announcement regarding RIO2016 and Zika on day 149, the WHO-Zika signal also had a peak on day 149; WHO/Zika–related tweets comprised 34% of all Zika-related tweets (Figure 4). These results supported H2 that Zika-related tweeting dynamics were triggered by other events in the real world.

#### Association Between Web-Based Influentials and Zika-Related Tweeting Dynamics

In this section, we present the role of TT, TR, TRRT, and TM influential user groups, as defined previously.

#### Comparison Between Each Group of Influentials and Zika-Related Tweeting Dynamics

**Figure 6:**
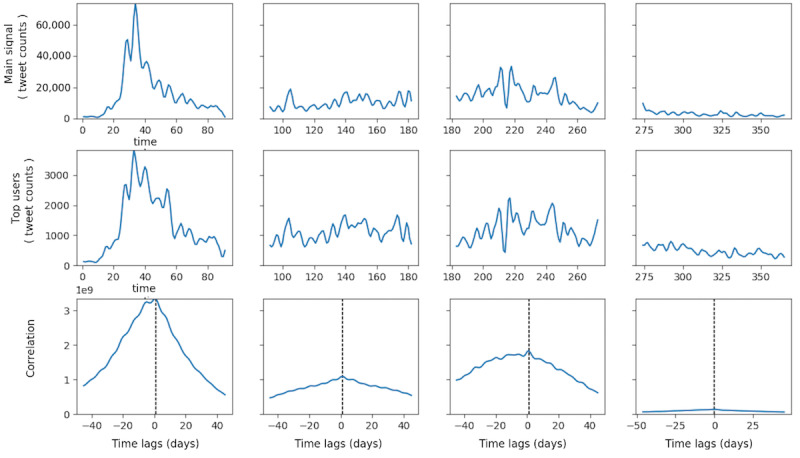
Quarterly correlation between the main Zika-related tweeting signals and users’ tweeting signals in the TT group. TT: top tweeter.

**Figure 7:**
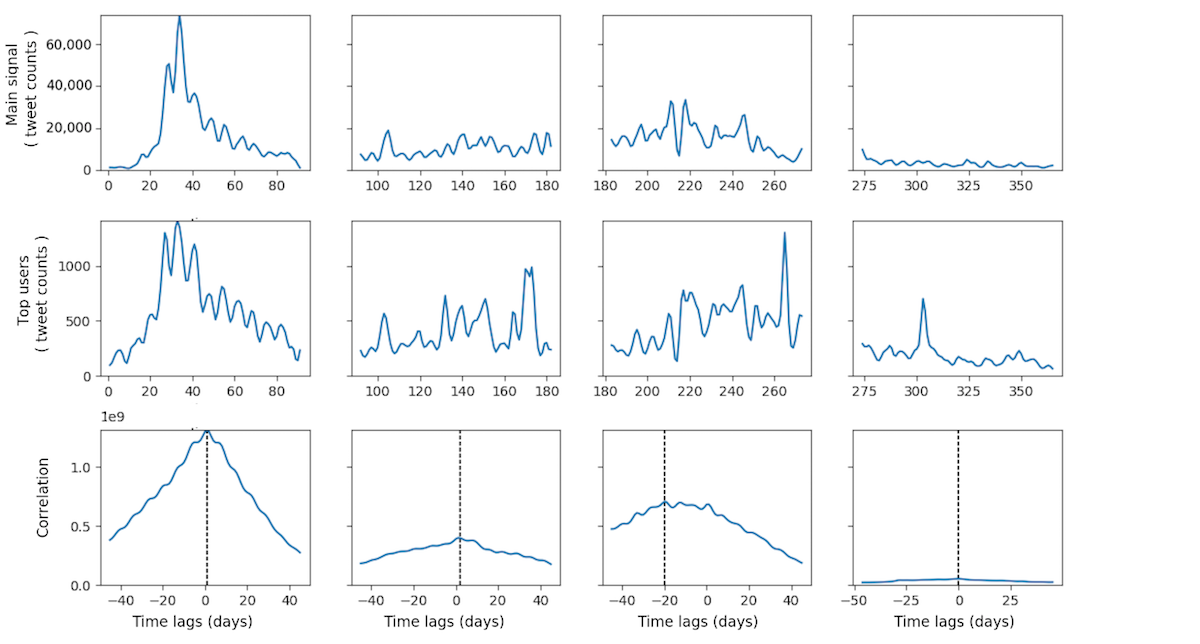
Quarterly correlation between the main signal and users in the TR group. TR: top retweeter.

**Figure 8:**
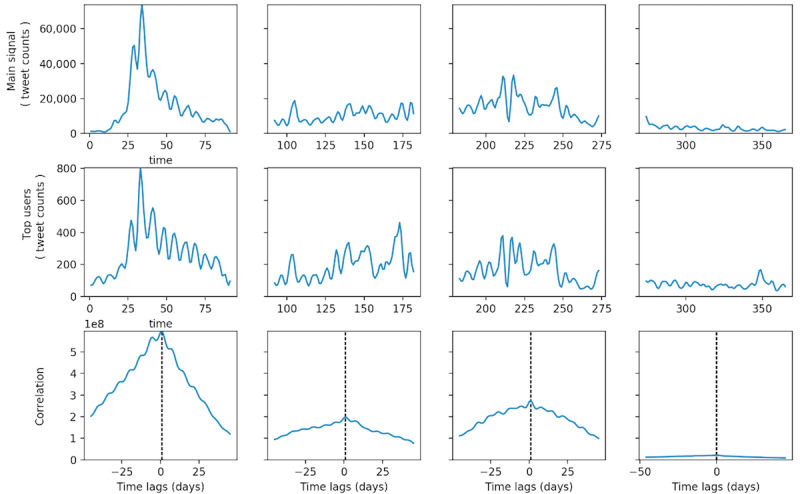
Quarterly correlation between the main signal and users in the TRRT group. TRRT: top received retweets.

**Figure 9:**
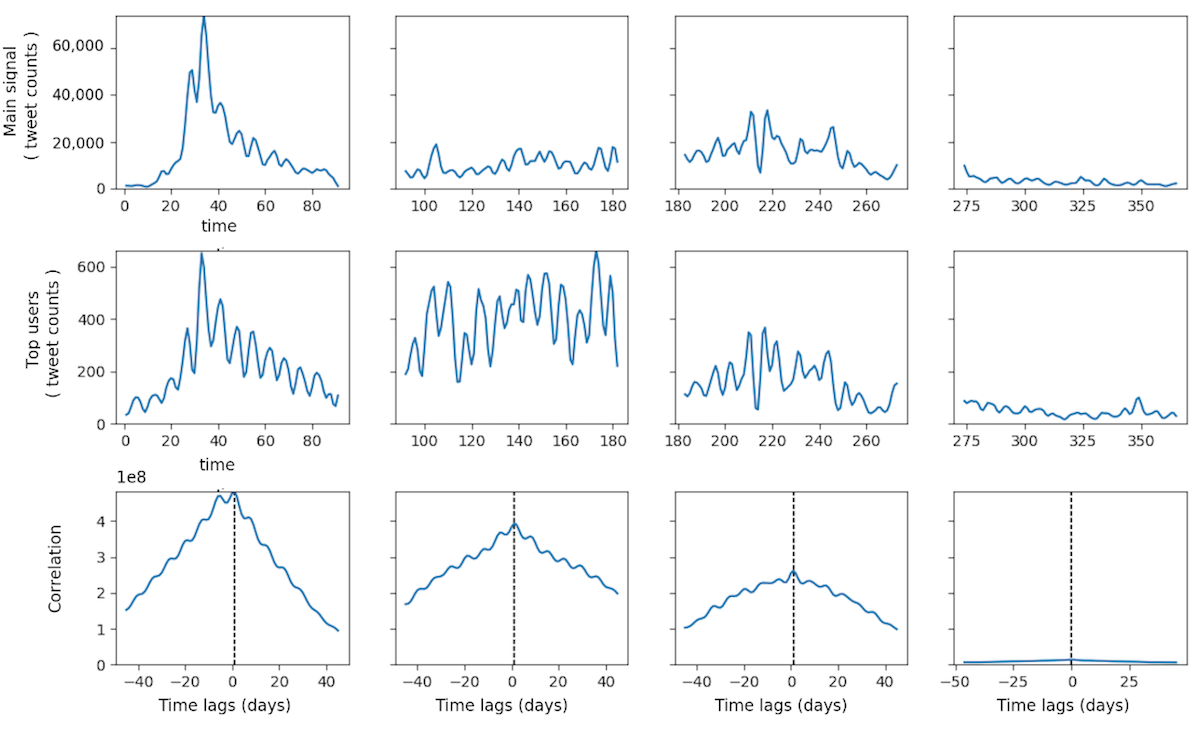
Quarterly correlation between the main signal and users in the TM group. TM: top mentioned.

Tweeting dynamics in the TRRT, TT, and TM groups and retweeting dynamics in the TR group were extracted and constructed for the top 100 users in each group. Quarterly association between these groups’ tweeting dynamics and overall Zika-related tweeting dynamics is shown in Figures 6-9. Each figure has 3 panels. The upper panel shows the overall tweeting dynamics, the middle panel demonstrates the tweeting dynamics of the particular influential group, and the bottom panel shows the CCF of the 2 signals. Group-level tweeting dynamics in TT, TM, and TRRT groups were highly correlated with and approximated the shape of the overall tweeting dynamics (Figures 6-8). However, the retweeting dynamics of the TR group were not closely associated with the overall Zika-related tweeting dynamics (Figure 9). In the TR group, there were peaks in their retweeting signal on days 170, 173, 265, and 303; however, no noticeable corresponding peaks were identified around these days in the main Zika-related tweeting signal. We conjectured that the TR group, in general, would be more active following certain undetected events, which did not necessarily coincide with the overall Zika-related tweeting dynamics.

More importantly, for TT, TRRT, and TM groups, the maximum CCF occurred at +1 day lag in the first three quarters of 2016 (Figures 6, 8, and 9), indicating that these groups’ tweeting activities were 1 day ahead of the overall discussion on Twitter. For example, the peaks in the overall Zika-related tweeting signal lagging behind the peaks in the TM group by approximately 1 to 2 days. Therefore, these influential groups’ tweeting activities were not only highly associated with the overall tweeting dynamics but these influentials were also the potential driving forces of the overall Zika discussions on Twitter. As a result, by observing a few hundred influentials’ tweeting activities, we could accurately predict the upcoming rise and fall in the overall tweeting dynamics. Nevertheless, this lag diminished to zero in the fourth quarter for all 3 influential groups, as the Zika epidemic and PHEIC ended in the fourth quarter of 2016.

**Figure 10:**
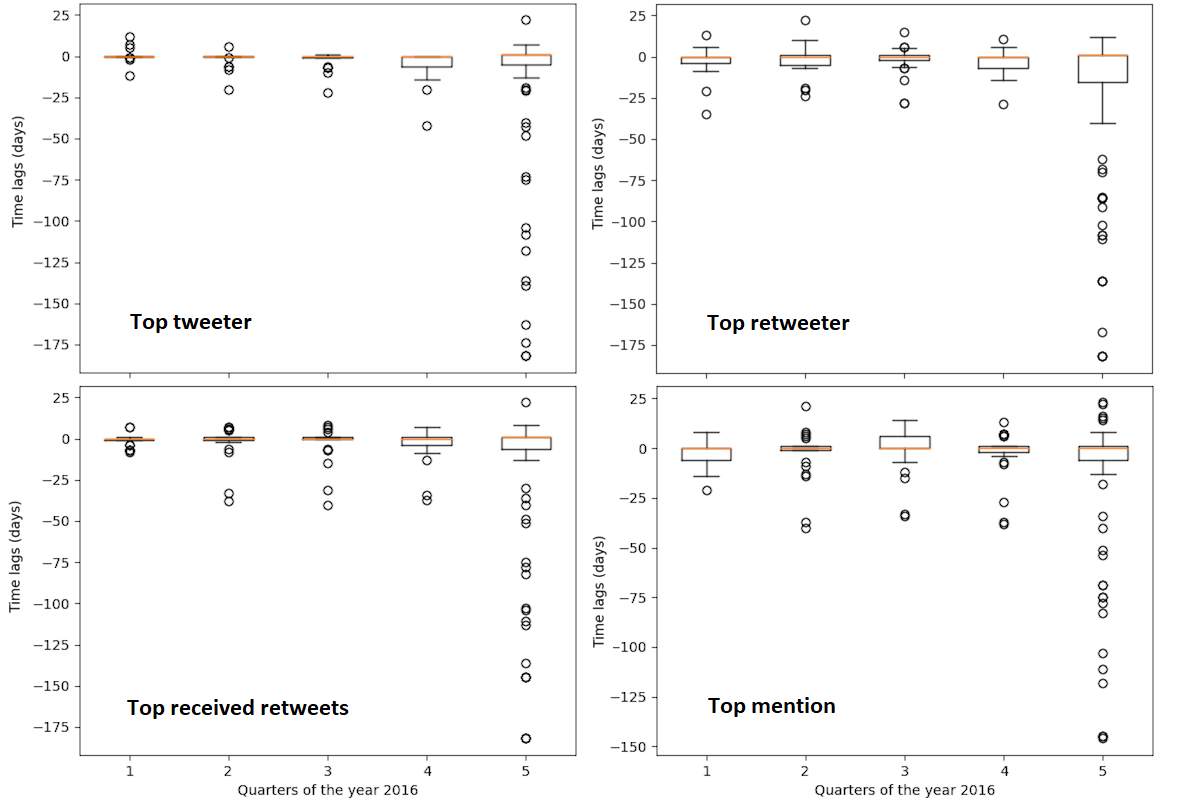
Comparison of tweeting activity of an individual user in 4 influential groups with the main signal.

In addition, we examined the contributions of individual users in each of these influential groups, TT, TRRT, and TM. We further calculated the CCF between a user’s tweeting time series and the overall tweeting dynamics in each quarter as well as in 2016 (Figure 10). Time lags of the majority of influential users were very close to zero, which implies that these users could not be driving the overall discussion of Zika on Twitter, but rather participating in the discussion. However, there were a few users whose time lags were substantially positive, indicating their potential role in driving the overall Zika-related tweeting dynamics. Furthermore, the quarterly results revealed the tweeting dynamics of influentials at a finer temporal resolution than yearly results (Figure 10). Note that in each panel, the first 4 boxplots (labels 1-4 on the x-axis) were quarterly, and the last one (label 5) was for the entire year of 2016. In general, most influentials did not engage in Zika discussions on Twitter constantly and continuously across the entire year of 2016. They might be active and highly influential during certain periods when they were interested in Zika and, hence, participated in discussions on Twitter. As a result, aggregating all individual influential users’ tweeting activities in the entire year would undermine each user’s temporal dynamics of tweeting and, consequently, its time-specific influence on the overall discussion dynamics on social media.

#### Overlap Between Influential Groups

In addition to exploring each potential influential group’s role in driving the Zika-related tweeting dynamics, we also investigated if different influential groups had overlaps. Table 2 shows the year-long intersections between any 2 groups of influentials, whereas Table 3 shows the overlap for selected groups on a quarterly basis. The TM group had no member who also belonged to the TM or TRRT groups, and the TT group had no intersection with the TRRT group. These results suggested that being highly active did not necessarily guarantee to receive a lot of mentions and/or RTs from other users on social media. Therefore, active and passive influentials discussing Zika on social media were distinctive users.

On a quarterly basis, there were quite a few influentials who were being mentioned and retweeted extensively at the same time (Table 3, column 2). On the other hand, there were only a few users in the TR group who were also highly mentioned and retweeted (Table 3, columns 1 and 3). These user accounts belonged to health organizations, such as @cdchep and @CDCChronic, and also a few well-known but independent individuals, such as @Laurie_Garrett and @MackayIM. This reinforced our previous finding that active and passive influentials were not the same users. For public health agencies such as the CDC, although they might actively disseminate information to the public on social media, their efforts were not well recognized by the general public users. Therefore, health agencies need to craft more effective strategies to engage public participation and discussion of an emerging health issue on social media.

**Table 2:** Overlap between the 4 groups of influentials in the entire 2016 period.

Influentials	TM^a^, n	TRRT^b^, n	TT^c^, n	TR^d^, n
TM	N/A^e^	47	11	0
TRRT	47	N/A	0	0
TT	11	0	N/A	6
TR	0	0	6	N/A

^a^TM: top mentioned.

^b^TRRT: top received retweets.

^c^TT: top tweeter.

^d^TR: top retweeter.

^e^N/A: not applicable.

**Table 3:** Quarterly overlap between selected influential groups.

Quarter in 2016	TM^a^-TR^b^	TM-TRRT^c^	TR-TRRT
1	4	49	3
2	4	43	5
3	3	44	2
4	6	45	8

^a^TM: top mentioned.

^b^TR: top retweeter.

^c^TRRT: top received retweets.

## Discussion

### Principal Findings and Future Work

Communicating with the general public is essential in risk communication during public health emergencies [37]. Hosting a large and diverse population, social media platforms such as Twitter are valuable resources for public health professionals to understand and analyze public opinions on emerging health issues [23,38-41]. During the 2016 Zika epidemic, Twitter was demonstrated to be an ideal place to explore public concerns and interests about the disease through time and across different locations [23,25,27,42-44]. In addition as a means of understanding public opinions, social media platforms are utilized by health professionals to communicate with the public and disseminate accurate and timely information regarding an ongoing health emergency [45]. For example, our previous study evaluated the role of CDC in disseminating Zika-related information on Twitter during the Zika outbreak. We revealed that the CDC played a critical role in tweeting Zika-related information during the first quarter of 2016 when the actual disease counts were still relatively low. However, the CDC’s Zika-related tweets quickly and drastically decreased after the first quarter of 2016, when the Zika case counts increased [27]. One important yet underexplored aspect of web-based discussions of health emergencies is to identify potential driving forces that can lead and change the dynamics of discussions on social media. Identifying such influential factors/contributors is critical for devising effective strategies in health crisis management and risk communication. Studies have shown that monitoring discussions on social media or search queries through infodemiology and infoveillance methods can help estimate or predict disease burden [28,31,46,47]. However, it is unclear if and how the actual situation of a health issue influences the public’s perception and discourse on social media. Moreover, the correlation between real-world events and their potential impact on web-based discussions of health emergencies is not well investigated and understood. In addition to investigating the impact of *what happened*, it is critical to evaluate the role of web-based influential actors, that is, those who would be web-based opinion leaders who drive web-based discussions.

These 3 research questions correspond to the 3 hypotheses investigated in this study. Our systematic and comprehensive analyses have provided a novel and holistic view of different factors impacting discussions about a health emergency on social media. This new perspective will help us better understand the complexity of such discussions.

In the future, there are a number of directions that we could pursue to further improve and expand this work. As an example, the last hypothesis that investigates the role of web-based influentials is not mutually exclusive from the first 2 hypotheses. For instance, our preliminary study has shown that during and immediately after the WHO-PHEIC announcement on February 1, 2016, many news agencies’ Twitter accounts helped disseminate this announcement on Twitter. Therefore, both the critical real-world event (WHO-PHEIC announcement) and web-based influentials (news agencies’ accounts) simultaneously drove Zika-related tweeting dynamics. In the future, we plan to further explore changes in the dynamics of discussions by constituent contributors.

In this study, we demonstrated a high association and temporal precedence between the tweeting activity of influentials and overall tweeting dynamics. The web-based tweeting signals of influentials preceded the overall tweeting signal regarding Zika, which were strong indicators of potential causality. Our results suggest that the tweeting activities of the TRRT, TT, and TM groups are good representatives of the overall tweeting dynamics. Therefore, their tweeting dynamics can be used to accurately approximate overall discussion dynamics on social media and to further predict the upcoming changes in discussion dynamics effectively.

To investigate discussion dynamics on Twitter, a highly sophisticated and complicated social media platform with millions of tweets, we utilized an array of different computational methods, including a time series analysis, signal processing, a content analysis, and information theory computations. In particular, we developed an analytical pipeline, EventPeriscope, to integrate and consolidate these different computations. The EventPeriscope pipeline is the practical outcome and contribution of this study. Compared with other similar analytical frameworks, EventPeriscope has the advantage of detecting both planned and unplanned events related to a specific discussion topic. This analytical pipeline can be readily transferred and applied to investigate other emerging or nonemerging issues on social media, such as the general discussion of health issues, identifying potential driving forces of the discussion, and evaluating their influence.

It should be noted that the 3 major drivers on tweeting dynamics mentioned in this study are not an exhaustive list of possible drivers. Further potential drivers, such as individual users or organization users and verified or unverified user status, will also be investigated in the future. In addition, we can also use EventPeriscope to detect other concurrent issues that might also influence Zika-related tweeting dynamics, such as the 2016 US presidential election. In addition, Zika case counts outside the United States could also be a potential driver, especially web-based discussions in Spanish and Portuguese.

### Conclusions

This study analyzed Zika-related tweeting dynamics in 2016 when Zika became a global concern. We revealed the potential drivers of Zika discussions on social media by testing 3 hypotheses. First, we demonstrated that Zika-related tweeting dynamics, that is, the time series of the daily number of Zika-related tweets, were not substantially associated with the underlying Zika epidemic (the time series of downscaled daily case counts) in the United States in any of the four quarters in 2016 as well as in the entire 2016 period. We then showed that peaks of Zika-related tweeting dynamics were significantly influenced by and associated with critical real-world events, both planned, such as the Rio Olympics, and unplanned, such as the WHO-PHEIC announcement. We further evaluated the role of potential web-based influentials and demonstrated that the TT, TM, and top users whose tweets were retweeted many times (TRRT) groups were potential drivers of the overall discussion of Zika on Twitter. Through these careful analyses of tweeting dynamics, our study revealed the potential contributors and drivers of a discussion on an emerging health topic. Insights gained from this study could be applied to other emerging health topics in the future. More importantly, we demonstrated the feasibility of our comprehensive analytical approach and the EventPeriscope framework to investigate web-based discussion dynamics of health emergencies and to identify the potential driving forces of these discussions.

## Abbreviations

CA: content analyzer
CCF: cross-correlation function
CDC: Centers for Disease Control and Prevention
DSI: Data Science Initiative
MI: mutual information
PHEIC: Public Health Emergency of International Concern
PMI: pointwise mutual information
Regex: regular expression
RTs: retweets
TM: top mentioned
TR: top retweeter
TRRT: top received retweets
TT: top tweeter
WHO: World Health Organization

## References

[1] Alshaikh F, Ramzan F, Rawaf S, Majeed A (2014). Social network sites as a mode to collect health data: a systematic review. J Med Internet Res.

[2] Balatsoukas P, Kennedy CM, Buchan I, Powell J, Ainsworth J (2015). The role of social network technologies in online health promotion: a narrative review of theoretical and empirical factors influencing intervention effectiveness. J Med Internet Res.

[3] Charles-Smith LE, Reynolds TL, Cameron MA, Conway M, Lau EH, Olsen JM, Pavlin JA, Shigematsu M, Streichert LC, Suda KJ, Corley CD (2015). Using social media for actionable disease surveillance and outbreak management: a systematic literature review. PLoS One.

[4] Colditz JB, Chu K, Emery SL, Larkin CR, James AE, Welling J, Primack BA (2018). Toward real-time infoveillance of Twitter health messages. Am J Public Health.

[5] Grajales FJ, Sheps S, Ho K, Novak-Lauscher H, Eysenbach G (2014). Social media: a review and tutorial of applications in medicine and health care. J Med Internet Res.

[6] Hamm MP, Chisholm A, Shulhan J, Milne A, Scott SD, Given LM, Hartling L (2013). Social media use among patients and caregivers: a scoping review. BMJ Open.

[7] Hu Y (2015). Health communication research in the digital age: a systematic review. J Commun Healthc.

[8] Kass-Hout TA, Alhinnawi H (2013). Social media in public health. Br Med Bull.

[9] Lardon J, Abdellaoui R, Bellet F, Asfari H, Souvignet J, Texier N, Jaulent M, Beyens M, Burgun A, Bousquet C (2015). Adverse drug reaction identification and extraction in social media: a scoping review. J Med Internet Res.

[10] Maher CA, Lewis LK, Ferrar K, Marshall S, de Bourdeaudhuij I, Vandelanotte C (2014). Are health behavior change interventions that use online social networks effective? A systematic review. J Med Internet Res.

[11] Moorhead SA, Hazlett DE, Harrison L, Carroll JK, Irwin A, Hoving C (2013). A new dimension of health care: systematic review of the uses, benefits, and limitations of social media for health communication. J Med Internet Res.

[12] Neiger BL, Thackeray R, van Wagenen SA, Hanson CL, West JH, Barnes MD, Fagen MC (2012). Use of social media in health promotion: purposes, key performance indicators, and evaluation metrics. Health Promot Pract.

[13] Sarker A, Ginn R, Nikfarjam A, O'Connor K, Smith K, Jayaraman S, Upadhaya T, Gonzalez G (2015). Utilizing social media data for pharmacovigilance: a review. J Biomed Inform.

[14] Sinnenberg L, Buttenheim AM, Padrez K, Mancheno C, Ungar L, Merchant RM (2017). Twitter as a tool for health research: a systematic review. Am J Public Health.

[15] Sloane R, Osanlou O, Lewis D, Bollegala D, Maskell S, Pirmohamed M (2015). Social media and pharmacovigilance: a review of the opportunities and challenges. Br J Clin Pharmacol.

[16] Velasco E, Agheneza T, Denecke K, Kirchner G, Eckmanns T (2014). Social media and internet-based data in global systems for public health surveillance: a systematic review. Milbank Q.

[17] Webb TL, Joseph J, Yardley L, Michie S (2010). Using the internet to promote health behavior change: a systematic review and meta-analysis of the impact of theoretical basis, use of behavior change techniques, and mode of delivery on efficacy. J Med Internet Res.

[18] Hasan M, Orgun MA, Schwitter R (2018). A survey on real-time event detection from the Twitter data stream. J Inf Sci.

[19] Dredze M, Broniatowski DA, Hilyard KM (2016). Zika vaccine misconceptions: a social media analysis. Vaccine.

[20] Jamison AM, Broniatowski DA, Quinn SC (2019). Malicious actors on Twitter: a guide for public health researchers. Am J Public Health.

[21] Valentini C (2015). Is using social media 'good' for the public relations profession? A critical reflection. Public Relat Rev.

[22] Hadi TA, Fleshler K (2016). Integrating social media monitoring into public health emergency response operations. Disaster Med Public Health Prep.

[23] Khatua A, Khatua A (2016). Immediate and Long-Term Effects of 2016 Zika Outbreak: A Twitter-Based Study. Proceedings of the 18th International Conference on e-Health Networking, Applications and Services.

[24] Fu K, Liang H, Saroha N, Tse ZT, Ip P, Fung IC (2016). How people react to Zika virus outbreaks on Twitter? A computational content analysis. Am J Infect Control.

[25] Glowacki EM, Lazard AJ, Wilcox GB, Mackert M, Bernhardt JM (2016). Identifying the public's concerns and the Centers for Disease Control and Prevention's reactions during a health crisis: an analysis of a Zika live Twitter chat. Am J Infect Control.

[26] Lowe R, Barcellos C, Brasil P, Cruz O, Honório NA, Kuper H, Carvalho M (2018). The Zika virus epidemic in Brazil: from discovery to future implications. Int J Environ Res Public Health.

[27] Chen S, Xu Q, Buchenberger J, Bagavathi A, Fair G, Shaikh S, Krishnan S (2018). Dynamics of health agency response and public engagement in public health emergency: a case study of CDC tweeting patterns during the 2016 Zika epidemic. JMIR Public Health Surveill.

[28] Masri S, Jia J, Li C, Zhou G, Lee M, Yan G, Wu J (2019). Use of Twitter data to improve Zika virus surveillance in the United States during the 2016 epidemic. BMC Public Health.

[29] Avery EJ (2017). Public information officers’ social media monitoring during the Zika virus crisis, a global health threat surrounded by public uncertainty. Public Relat Rev.

[30] Hadi T, MacGregor J, Mann L (2017). Social media monitoring: 2016 Zika response in NYC. Health Security.

[31] Eysenbach Gunther (2009). Infodemiology and infoveillance: framework for an emerging set of public health informatics methods to analyze search, communication and publication behavior on the Internet. J Med Internet Res.

[32] Hall V, Walker WL, Lindsey NP, Lehman JA, Kolsin J, Landry K, Rabe IB, Hills SL, Fischer M, Staples JE, Gould CV, Martin SW (2018). Update: noncongenital Zika virus disease cases-50 US States and the district of Columbia, 2016. MMWR Morb Mortal Wkly Rep.

[33] Newman D, Lau JH, Grieser K, Baldwin T (2010). Automatic Evaluation of Topic Coherence. Proceedings of the 2010 Conference of the North American Chapter of the Association for Computational Linguistics: Human Language Technologies.

[34] Nikolaos P, Ioannis K, Dimitrios G, Michaelis S, Piatkowski N, Stolpe M (2016). Detecting events in online social networks: definitions, trends and challenges. Solving Large Scale Learning Tasks. Challenges and Algorithms: Essays Dedicated to Katharina Morik on the Occasion of Her 60th Birthday.

[35] (2016). World Health Organization.

[36] Attaran A (2016). Harvard Public Health Review.

[37] Peter B, Kenneth C, Sarah C, Denis FS (2010). Risk Communication and Public Health.

[38] Miller M, Banerjee T, Muppalla R, Romine W, Sheth A (2017). What are people tweeting about Zika? An exploratory study concerning its symptoms, treatment, transmission, and prevention. JMIR Public Health Surveill.

[39] Paul MJ, Dredze M (2017). Social monitoring for public health. Synth Lect Info Concepts Retrieval Serv.

[40] Rezaallah B, Lewis DJ, Pierce C, Zeilhofer HF, Berg BI (2019). Social media surveillance of multiple sclerosis medications used during pregnancy and breastfeeding: content analysis. J Med Internet Res.

[41] Southwell BG, Dolina S, Jimenez-Magdaleno K, Squiers LB, Kelly BJ (2016). Zika virus-related news coverage and online behavior, United States, Guatemala, and Brazil. Emerg Infect Dis.

[42] Daughton AR, Paul MJ (2019). Identifying protective health behaviors on Twitter: observational study of travel advisories and Zika virus. J Med Internet Res.

[43] Farhadloo M, Winneg K, Chan MS, Hall Jamieson K, Albarracin D (2018). Associations of topics of discussion on Twitter with survey measures of attitudes, knowledge, and behaviors related to Zika: probabilistic study in the United States. JMIR Public Health Surveill.

[44] Stefanidis A, Vraga E, Lamprianidis G, Radzikowski J, Delamater PL, Jacobsen KH, Pfoser D, Croitoru A, Crooks A (2017). Zika in Twitter: temporal variations of locations, actors, and concepts. JMIR Public Health Surveill.

[45] Guidry JP, Jin Y, Orr CA, Messner M, Meganck S (2017). Ebola on Instagram and Twitter: how health organizations address the health crisis in their social media engagement. Public Relat Rev.

[46] Eysenbach Gunther (2006). Infodemiology: tracking flu-related searches on the web for syndromic surveillance. AMIA Annu Symp Proc.

[47] Chew Cynthia, Eysenbach Gunther (2010). Pandemics in the age of Twitter: content analysis of Tweets during the 2009 H1N1 outbreak. PLoS One.

